# Multiplex detection of bacteria on an integrated centrifugal disk using bead-beating lysis and loop-mediated amplification

**DOI:** 10.1038/s41598-017-01415-x

**Published:** 2017-05-03

**Authors:** He Yan, Yunzeng Zhu, Yan Zhang, Lei Wang, Junge Chen, Ying Lu, Youchun Xu, Wanli Xing

**Affiliations:** 1 Department of Biomedical Engineering, School of Medicine, Tsinghua University, Beijing, 100084 China; 2 Collaborative Innovation Center for Diagnosis and Treatment of Infectious Diseases, Hangzhou, 310003 China; 3 National Engineering Research Center for Beijing Biochip Technology, Beijing, 102206 China

## Abstract

Although culture-based identification of bacteria is the gold-standard for the diagnosis of infectious diseases, it is time consuming. Recent advances in molecular diagnostics and microfluidic technologies have opened up new avenues for rapid detection of bacteria. Here, we describe a centrifugal-microfluidic chip for the detection of bacteria by integrating the cell lysis, clarification, and loop-mediated amplification (LAMP). The major advantages of this chip are as follows. Firstly, bacteria lysis was innovatively achieved by rotating a pair of magnets to generate bead-beating while the chip was kept stationary during lysis, which simplified the chip design because no additional valve was needed. Secondly, the on-chip assay time was short (within 70 min), which was competitive in emergency situations. Thirdly, results of the analysis can be interpreted by using a fluorescence detector or by the naked-eye, making it versatile in many areas, especially the resource-limited areas. The on-chip limits of detection of six types of bacteria were valued by gel electrophoresis, showing the similar results compared to the bench-top LAMP protocol. This chip can be used for rapid, sensitive, accurate and automated detection of bacteria, offering a promising alternative for simplifying the molecular diagnostics of infectious diseases.

## Introduction

Bacterial infections pose a major threat to global health. Each year, diseases like meningitis, pneumonia, and sepsis caused by bacteria continue to be the cause of numerous deaths globally^1–3^. Early identification of the type of bacteria responsible for the manifestation of the disease is vital to prevent complications arising out of the progression of the disease and is beneficial for formulating an effective therapy for treating patients. Traditionally, identification and counting of bacterial pathogens in clinical samples have relied heavily on culture-based methods. These methods are time-consuming and usually require 24 h to several days for the completion of the analysis^4^. Hence, many therapies are conducted empirically on patients without any prior knowledge of the identity of the causative agent. This has lead to the unnecessary use, misuse, or abuse of antimicrobials and has in many cases worsened the patient’s condition with an increased risk of mortality^5, 6^.

Recently, techniques like polymerase chain reaction (PCR) and loop-mediated amplification (LAMP) that can amplify specific regions of nucleic acids (NA) have been adopted for the detection of microbes. They offer benefits like speed, precision and increased sensitivity when compared to the culture-based diagnostics^7–10^. Consequently, numerous commercial kits for detection of microbes based on PCR or LAMP-assisted amplification of microbial NA (DNA or RNA) have entered the markets in recent years. Although these methods have enabled a dramatic reduction in the time required for the identification of the pathogen^7^, they involve cumbersome protocols for sample preparation as well as NA amplification and detection. Furthermore, the instrumentation used for performing the analysis is expensive and requires well-trained personnel for performing the various steps of the analysis^11^. Another serious concern about employing highly specific amplification methods like LAMP and nested PCR is the possibility of a false positive result arising out of extraneous NA contamination^12, 13^. These drawbacks have hindered the widespread use of such techniques in routine clinical diagnostics. A possible and effective solution to overcoming these limitations would be to integrate the different steps of analysis into a miniaturized and automated device.

Ideally, a device for the detection of bacteria should perform all the steps, including cell lysis, DNA extraction, amplification, and detection, in an integrated and automated manner to facilitate a simplified “sample-in to answer-out” detection. Some pioneering work has already been performed towards achieving this goal, for example, Czilwik *et al*.^14^ have shown that the steps of chemical lysis, DNA extraction, and nested PCR can be integrated successfully into a single centrifugal microfluidic disk. In their work, different types of bacteria that were spiked in the serum were first lysed by using chemical reagents. The subsequent steps of binding, washing and elution of DNA were conducted by employing magnetic beads. Finally, the purified DNA was amplified and detected by using nested real-time PCR. The disk assay achieved a satisfactory limit of detection (LoD) for *Streptococcus agalactiae*, *Staphylococcus warneri*, *Escherichia coli*, and *Haemophilus influenza*. But the turnaround time of the complete analysis was up to 3 h and 45 min. Another integrated chip developed for detection of bacteria by Roy *et al*.^15^ incorporated mechanical cell lysis, PCR, amplicon digestion, and microarray hybridization steps. The time required to perform the analysis was 2 h and 10 min. The device could successfully detect spores of *Bacillus atropheus*. However, the presence of a single PCR chamber hampers multiplexing by this device. Hwang *et al*.^16^ demonstrated the advantage of having a miniaturized bead-beating device for automation of NA extraction from Gram-positive bacteria. Bead-beating was actuated by the pneumatic vibration of a polydimethylsiloxane (PDMS) membrane. Using such a strategy, *Staphylococcus aureus* could be detected successfully. However, the use of glass-based material as well as pumps/valves in the system increased the complexity of the device for fabrication and its use. Boehm *et al*.^17^ utilized impedance-based measurement and antibodies to detect and identify bacteria. But the sensitivity of the method was lower when compared to PCR and LAMP-based methods.

More recently, commercial products with simple workflows have also been introduced in the markets. The GeneXpert platform^18^ from Cepheid integrates different analytical steps into a single cartridge for the detection of bacteria or viruses, such as Methicillin-resistant *S. aureus*, *Clostridium difficile*, and Norovirus, but each cartridge can only detect one pathogen. Another product named FilmArray^19^ from BioFire Diagnostics uses nested PCR and enables the multiplexed detection of pathogens, but it is not fully integrated. An additional device is needed to accomplish sample loading. Furthermore, the apparatus is relatively more expensive when compared to other methods available for detection of pathogens.

Our goal was to develop an automated and user-friendly device to enable multiplexed detection of bacteria quickly and precisely. Several considerations were taken into account while constructing a prototype of the device. The first consideration was the selection of the method for regulating the flow of the liquids in the chip. Flow of liquids in microfluidics-based systems can be regulated by using a system actuated by pumps and valves^20, 21^ or a system actuated by centrifugation^22^. Precise and complex control of liquid can be achieved using pumps and valves, but the connection of chips to pumps is usually not user-friendly. In addition, there is a risk of potential NA contamination during the assembly of the device. Further, the volume of a valve or pump based system cannot be conveniently miniaturized. A centrifugal-microfluidic platform offers an efficient alternative for integrating all the steps of the analysis on a disk, and the chip can be fully sealed after the injection of the sample, lowering the risk of contamination by exogenous NAs. Moreover, all the fluidic operations can be accomplished by using a single rotary motor, which enables the construction of a portable and easy-to-use device.

The second consideration involved the selection of method for the lysis of bacteria. Cell lysis is an important step during the analysis and has a profound impact on the success of the diagnosis. Cell walls of Gram-positive bacteria differ significantly from those of Gram-negative organisms and therefore the efficiency of lysis for these bacteria differs^23^. Cells can be lysed by either using chemical or physical methods. Chemical methods utilize enzymes and chaotropic salts to disrupt cell walls and cell membranes^24, 25^. Purified DNA can then be obtained by DNA binding, washing, and elution. Chemical lysis requires the use of different enzymes for lyzing different types of bacteria. For example, lysozyme is enough for disrupting the cell walls of commonly found Gram-positive bacteria such as *Bacillus subtilis* and lyses the cells. However, for lyzing *S. aureus* cells, which have a much thicker cell wall, at least two enzymes (lysozyme and lysostaphin) are needed^26^. Moreover, enzymatic reactions require temperature control and a long incubation time (~1–2 h). Reagents like alcohol and chaotropic salts could inhibit the subsequent amplification step if not eliminated completely. All these concerns make integration of chemical lysis on a chip challenging. In comparison, methods based on physical lysis of cells such as mechanical lysis^25, 27, 28^, thermal lysis^29^, laser lysis^30^, and sonolysis^31, 32^ are not only faster than enzymatic reactions, but also are equally efficient in disrupting cell walls of all types of microorganisms^33^. Among these methods, mechanical lysis by bead-beating is an effective way to lyse bacteria or fungi through impact force and shear effect^34, 35^. An off-chip device called OmmiLyse Bead Blender and the on-chip system designed by Siegrist *et al*.^34^ are examples of devices using this method for lysing bacterial cells. Zirconia bead is a common material for cell lysis and the bead itself is inert. Due to the relatively lower electrostatic effect compared to glass beads, it is easier for us to add the beads into the lysis chamber. Therefore, we chose zirconia bead-beating for lysing cells in our prototype for detection and identification of bacteria.

The third consideration was the selection of a technique for amplification of NAs. Typically, PCR runs require the reaction mixture to be maintained at three distinct temperatures during thermal cycling. The requirement for precise control of temperature increases the bulkiness and cost of the apparatus, which impedes the use of the device for on-site NA analysis^36^. Methods that do not require different temperature controls for amplification of NAs can be substituted for PCR. Isothermal amplification methods such as LAMP^36^, nucleic acid sequence-based amplification (NASBA)^37^, rolling circle amplification (RCA)^38^ and recombinase polymerase amplification (RPA)^39^ are widely used for pathogen detection and employ only one mild temperature to facilitate the reaction. For example, LAMP utilizes six primers and a DNA polymerase with strand displacement activity to perform reactions at 60–65 °C within 1 h^36^. The reaction takes only about 20 min for RPA at a constant temperature (37–40 °C)^39^, but it may lead to false positive results due to the relatively lower reaction temperature. In this work, LAMP was chosen for the integration and miniaturization of a NA analysis instrument.

After taking into account the above considerations, we have designed and developed a centrifugal-microfluidic disk for multiplexed detection of bacteria. Differing from other on-chip bead-beating methods actuated by stationary magnets and the requirement of high-speed centrifugation of the disk^15, 34^, we innovatively immobilized the chip and twirled the magnets with a motor in our prototype of the device. Upon actuation, the magnetic stirring bar in the lysis chamber made the beads collide with the bacteria. This decreased the times of high-speed rotation of the chip itself and simplified the design and integration of on-chip valves to stop the flow of the fluid under high-speed centrifugation. In our experiments, the real-time detection and end-point detection can be both achieved. The end-point detection can be done by fluorescent imaging or naked-eye observation. The limits of detection were tested by gel electrophoresis and real-time amplification. When compared to the work done by Czilwik *et al*.^14^, our design provided a more universal approach for bacteria lysis, which is suitable for both Gram-negative and Gram-positive bacteria without the concern of the types of lysis enzymes or the lysis protocol. Besides, the time required to perform the entire analysis using our method was about 3 times shorter (70 min vs. 225 min) and the protocol was simpler, making it more suitable for point-of-care testing. We optimized parameters like the volume of the beads, the rotation speed of the magnets and the time of lysis to determine the best conditions for the lysis of bacteria. The chip was experimentally tested for its ability to perform multiplexed detection of bacteria in mimics of clinical samples. The results of these experiments and the potential of the chip for use in clinical diagnostics for identification of bacteria to provide early and crucial information for physicians to conduct life-saving therapies are discussed.

## Materials and Methods

### Fabrication and architecture of the chip

**Figure 1:**
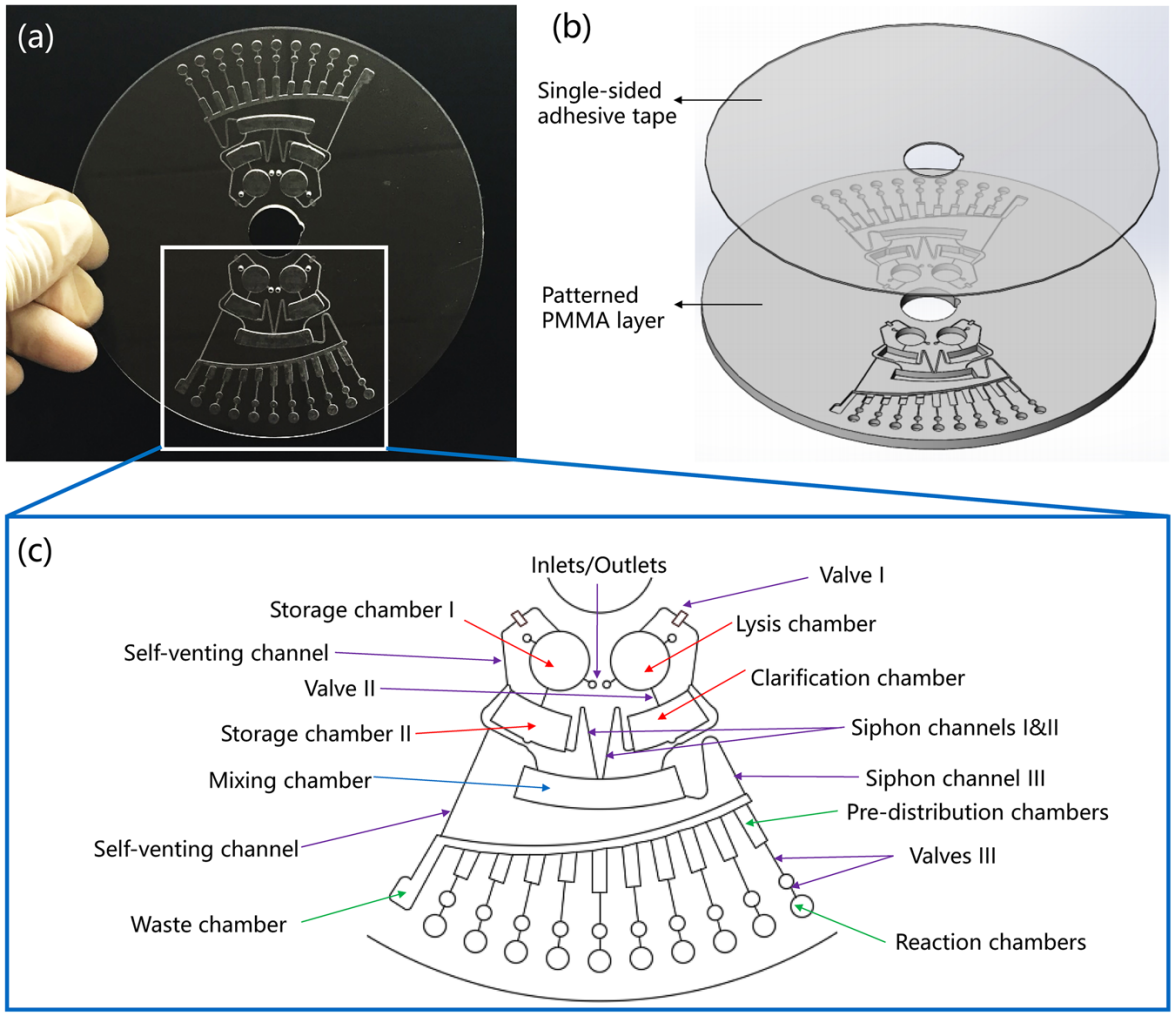
Schematic of the centrifugal chip for detection of bacteria. (**a**) Photo of the chip, which includes two parallel testing sections; (**b**) 3D illustration of the chip. (**c**) Each testing section comprises of four units: the lysis/clarification and storage unit, the mixing unit, the pre-distribution/reaction unit, and the liquid control unit, indicated by red, blue, green, and purple arrows, respectively.

Polymethyl methacrylate (PMMA) was used to make the disk. Each disk, measuring 60 mm (radius) × 3 mm (thickness), was fabricated by CNC milling (Hongyangchensheng Technology, Beijing, China). Disks consisted of a patterned PMMA layer and were sealed by using single-sided pressure-sensitive adhesives (PSA, Adhesive Research, Inc., Shanghai, China)^40^. Two to four identical sections could be assembled for performing multiple assays simultaneously. Each section consisted of a lysis/clarification and storage unit, a mixing unit, a pre-distribution/reaction unit, and a liquid control unit (Fig. 1 & Supplementary Fig. S1).

#### Lysis/clarification and storage unit

**Figure 2:**
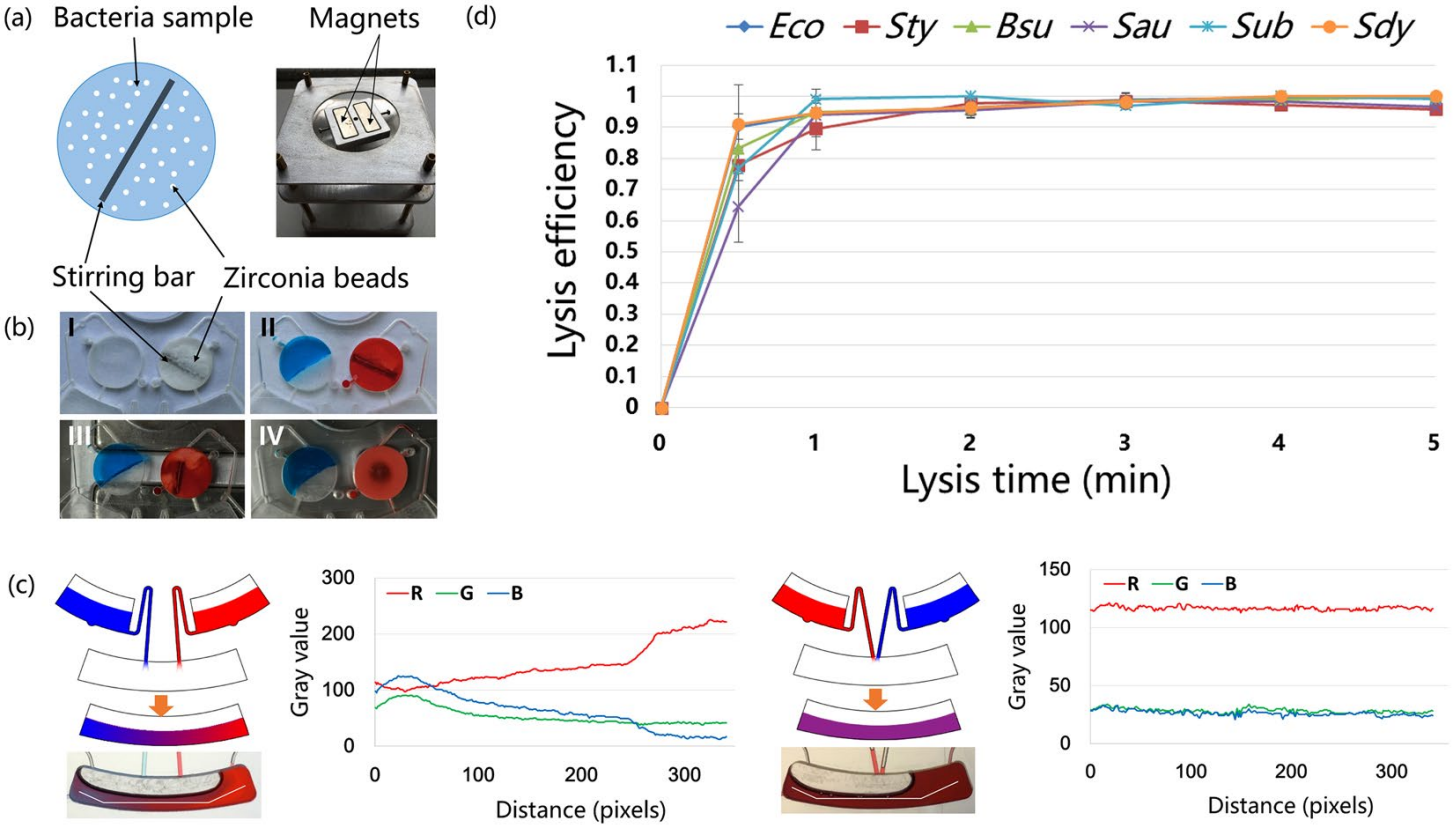
Illustration of lysis chamber, mixing chamber, and the optimal lysis time for six kinds of bacteria. (**a**) Illustration of the lysis chamber and the photo of the magnets equipped with a motor. (**b**) Photos of the lysis chamber, zirconia beads, and stirring bar. Picture I shows the filling of beads and bar, and then, sample (red dye) and mastermix (blue bye) were injected into the chip (II). Next, the chip was placed above the magnets (III), and a rotary motor was actuated (IV). (**c**) Two different configurations of the siphon channels. Radial siphon channels led to inhomogeneous mixing, but when the siphon channels were close to each other, uniform mixing was obtained. The white broken line in the mixing chamber displays the route of the RGB analysis. (**d**) Optimization of lysis time for different bacteria.

The lysis chamber was circular in shape with a diameter of 8 mm and a depth of 2 mm. The volume of the chamber was ~100 μL. The chamber consisted of a magnetizable stirring bar of 7 mm length and zirconia beads (~100 μm diameter, Zhimo New Material Technology, Shanghai, China) as illustrated in Fig. 2a and b. The rotation of the stirring bar was actuated by two rotating magnets (Fig. 2a), which set the beads in motions. Collision of the beads with bacteria resulted in the disruption of their cell walls and membranes. After lysis, the lysate was transferred to the clarification chamber via high-speed centrifugation. Cell debris precipitated during the process. The transfer of the LAMP master mix from storage chamber I to storage chamber II occurred simultaneously.

#### Mixing unit

Homogeneous mixing of the LAMP master mix with the lysate is important for the success of the amplification reaction. In our preliminary experiments, we found out that the position of the two siphon channels I and II affected the mixing of the two solutions. As shown in Fig. 2c, when the direction of the two siphon channels were radial, the lysate and master mix (represented by the blue and red colors, respectively) could not be mixed uniformly. However, relatively homogeneous mixing was achieved when the channels were brought closer to each other. RGB analysis by ImageJ software (NIH) supported the observed enhancement in mixing of the two liquids achieved by changing the position of the channels. We believe that the homogeneous mixing occurs due to the crossing of the paths of the two liquids such that the two solutions blend quickly during the liquid transfer. Though further mixing can be achieved by spinning the disk back and forth, this design can simplify the spin program.

#### Pre-distribution/reaction unit

After mixing, the mixture of the lysate and LAMP master mix was introduced into each reaction chamber. The volume of the reaction chamber was 10 μL, and specific primer pairs were preloaded into each well and dried under room temperature before sealing the chip. To prevent the cross-contamination of primers during liquid transfers, a pre-distribution unit (Fig. 1) was built in the device^14^, where the mixture was aliquoted into 10 pre-distribution chambers before it was centrifuged into the reaction chambers. After distribution of the reaction mixtures into the reaction chambers, LAMP was conducted.

#### Liquid control unit

In addition to centrifugal force, liquid control was enabled by capillary/hydrophobic valves, siphon channels, and self-venting channels. A sudden expansion/narrowing of the micro-channel traps the liquid meniscus^15, 22, 41^ (Supplementary Fig. S2) such that the lysate can be restricted to the lysis chamber by valves I and II under the normal pressure produced by rotation of the stirring bar until the spinning force overcomes the resisting capillary force of valve II. The valves were made hydrophobic by coating them with perfluorocarbon liquid EGC-1700 (3 M, China) to prevent unwanted priming of the capillary while the chip was at rest or bead-beating was being performed. 2% Tween 20 dissolved in ethanol was used to fill the siphon channels by a pipette. After the evaporation of the ethanol, Tween 20 was adsorbed on the surfaces to make the siphon channels hydrophilic. High centrifugal force maintained the meniscus front below the crest level of the siphon channel. Once the spinning speed was reduced below a certain value, the channel was primed by capillary force and the meniscus passed the crest point (Supplementary Fig. S2)^22^, this lead to the siphoning of the liquid into the chamber below. A self-venting channel was also necessary for gas circulation in the chip because the chip was fully sealed before the assay.

### Cell culture and counting

*E. coli* (Gram-negative, DH5α, TranGen Biotech., China), *B. subtilis* (Gram- positive, ATCC 6633), *Salmonella typhimurium* (Gram-negative, ATCC 14028), and *S. aureus* (Gram-positive, ATCC 6538) used in our experiments were grown in 20 mL Luria-Bertani (LB) broth (Oxoid, England) at 37 °C for 12 h. After growth, a small portion of the culture was diluted to an appropriate concentration with water, and the cell counts were estimated by plating diluted cultures on nutrient agar plates and counting the colonies formed. *Streptococcus uberis* (Gram-positive, ATCC 700407) and *Streptococcus dysgalactiae* (Gram-positive, ATCC 12388) were cultured and counted using Brain Heart Infusion (BHI) medium (AoBoXing Bio-Tech, China). The remaining liquid cultures were diluted with water to different concentrations for the actual on-chip experiments.

### Reagents and devices

The genomic DNAs (gDNAs) of bacteria used in the reference assays were extracted by chemical methods using a TIANamp Bacteria DNA Kit (Tiangen Biotech, Beijing, China). The concentrations of the purified gDNAs were calculated using a Nanodrop1000 spectrophotometer (ThermoFisher Scientific, USA) and diluted with water to desired concentrations. The 2 × LAMP mastermix was provided by CapitalBio Corporation (Beijing, China), and bovine serum albumin (BSA, final concentration of 3 mg mL^−1^) was added to the mastermix (total volume of 60 μL) to decrease the non-specific adsorption of enzymes^40^. Calcein, manganese chloride, and BSA were purchased from Sigma-Aldrich (Shanghai, China). The DL 2000 DNA marker was purchased from TaKaRa (Dalian, China), and GeneGreen dye was purchased from Tiangen Biotech (Beijing, China). The gel and chip images were obtained and processed using a gel imager (C150, Azure Biosystems, USA). All LAMP primer pairs were synthesized by Invitrogen (Beijing, China), and the sequences are listed in Supplementary Table S1. Each LAMP reaction required four to six primers named F3, B3, FIP, BIP, LF, and LB (LF and LB are not necessary), and the final concentration of each primer in our experiments was 0.3, 0.3, 2.4, 2.4, 1, and 1 μM, respectively.

### Preparation of bacterial samples for analysis

*S. aureus* and *S. typhimurium* were both spiked into human serum at varying final concentrations (10^5^ CFU mL^−1^, 10^4^ CFU mL^−1^ and 10^3^ CFU mL^−1^) to mimic clinical samples. Sterile syringe filters (13 mm Millex, Merck-Millipore, China) were used to isolate bacteria from the serum. In our experiments, 200 μL serum was first diluted with 800 μL water. The sample was then pushed steadily through a filter using a syringe. The membrane was washed with 1 mL water. Since the pore size of the filter was 0.22 μm, *S. aureus* and *S. typhimurium* could be easily captured by the filter and enriched on the membrane. To re-suspend the bacteria in solution, the syringe was pulled slowly and water was drawn up and through the filter as shown in Supplementary Fig. S3. Bacteria isolated from the serum were enriched in 100 μL water for on-chip analysis. The real-time fluorescence intensity was detected via a home-made real-time detector.

## Results

### Optimization of parameters for lysis of bacteria

Efficient lysis of bacteria is a pre-requisite for the success of all NA amplification procedures. Therefore, we first optimized the cell breakage step. Three parameters, stirrer voltage, bead quantity, and lysis time, were varied to determine the optimal conditions for cell lysis. Because the distance between the lysis chamber and magnets (~3 mm) was kept constant in our experiments, the stirrer voltage indirectly reflects the rotation speed of the stirring bar. So, voltage was selected as a variable. *E. coli* cells were lysed for 30 s at different voltages (0, 1, 2, 3, 4, and 5 V; the corresponding rotation speeds of the magnets were shown in Supplementary Fig. S4). The time required for lysis of bacteria was selected such that it was insufficient to lyse all the bacteria. This helped in grading the degree of lysis at different voltages. The efficiency of lysis was evaluated by plating the lysates on nutrient agar plates and counting the colonies formed. As shown in Supplementary Fig. S5, relatively high lysis efficiency was obtained at 4 V. Higher voltage (5 V) yielded good results but no obvious improvement, thus 4 V was selected for subsequent experiments.

The quantity of beads in the lysis chamber affects the efficiency of lysis. Inadequate number of beads may lead to insufficient collisions with the bacteria and reduce the overall efficiency of lysis. On the other hand, excess amount of beads may not only impede the spin of the stirring bar but also occupy too much space in the lysis chamber, reducing the space available for the bacterial solution to be lysed. We used different amounts of zirconia beads (0–0.3 g) to lyse *E. coli* cells for 30 s. 0.2 g beads gave the best lysis efficiency (Supplementary Fig. S6). At this mass, the zirconia beads occupied ~33% of the volume of the lysis chamber.

Having optimized the voltage and the amount of beads, we then sought to determine the optimal time for lysis of six different types of bacterial cells. Accordingly, *E.coli (Eco), B. subtilis (Bsu), S. typhimurium (Sty), S. aureus (Sau), S. uberis (Sub)*, and *S. dysgalactiae (Sdy)* were lyzed under identical conditions. Lysis efficiency was calculated by using the formula (N_original_ − N_alive_)/N_original_, where N_original_ is the number of live bacteria before lysis, and N_alive_ is the number of bacteria alive after the lysis. As shown in Fig. 2d, the lysis efficiencies of different bacteria calculated over a range of time points (0.5–5 min) varied, but tended to stabilize after 3 min. We chose 3 min as the time for lysis because the lysis efficiency reached almost 98% at this time point. Some bacteria survived the lysis step probably because they were located in the inlet/outlet and were sucked out along with the lysate, which was analyzed for enumeration of live bacteria by plate counting. The results of the bacterial cell lysis experiments demonstrated that the on-chip bead-beating procedure adopted by us was highly efficient in lysing both Gram-negative (*Eco, Sty*) as well as Gram-positive bacteria (*Bsu, Sau, Sub*, and *Sdy*). At the end of 0.5 min, the efficiency of lysis for *Eco* (Gram-negative) cells was 90.2%, which was higher than those observed for Gram-positive bacteria like *Bsu* (83.3%), *Sau* (64.6%), an*d Sub* (76.8%). This observation further supports the fact that Gram-positive bacteria have sturdier cell walls, which are comparatively harder to lyse.

### Workflow of the chip

**Figure 3:**
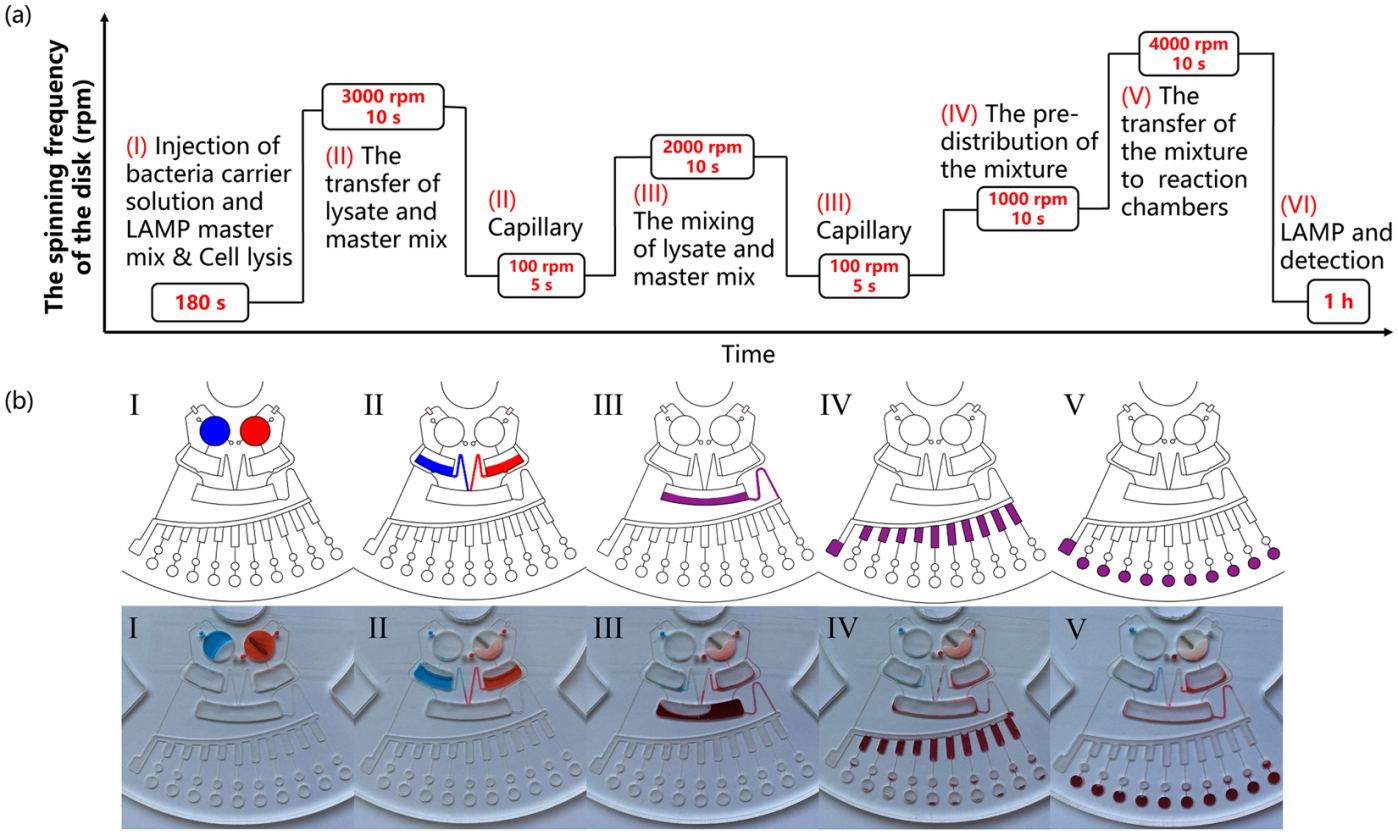
Spin protocol of the complete assay (**a**) and the corresponding illustrations and photos of each step (**b**).

Figure 3 illustrates the spin program and workflow with various spin frequencies and durations, which were controlled by a stepper motor in a custom-made instrument. Prior to initiating cell lysis, primer pairs were preloaded in each reaction chamber by using a pipette and dried at room temperature, in the future, the primer pairs can be preloaded by using a spotting machine (PersonalArrayer 16, CapitalBio, Beijing, China) to simplify the operation. Zirconia beads and the stirring bar were then placed in the lysis chamber. After surface coating of siphon channels and valves, the engraved side of the chip was sealed manually by using PSA. The sample (60 μL) and the mastermix (60 μL) were injected from the inlets located on the other side of the chip. After the injection, the inlets/outlets were also blocked by using PSA to obtain a fully sealed chip (Fig. 3a-I). Next, the chip was placed above the magnets, and cell lysis was conducted at 4 V for 3 min. A series of chip centrifugations were performed after the cell lysis step (see Supplementary video). First, the spin frequency was set to 3000 rpm for 10 s to burst open the valves. Lysate and LAMP mastermix got transferred into the clarification chamber and storage chamber II, respectively. During this step, cellular debris present in the lysate was precipitated and hence got separated, no DNA purification steps or concentration step is implemented compared to chemical methods, since we have conducted the off-chip process of sample pre-preparation, the amount of proteins and other impurities in the water solution was dramatically decreased. Next, the spin frequency was decreased to 100 rpm for 5 s so that the liquid could be primed by capillary action to fill the siphon channel (Fig. 3b-II). Then, the lysate and mastermix were transferred into the mixing chamber and uniformly mixed by applying centrifugal force at 2000 rpm for 10 s (Fig. 3b-III). Similar to the previous step, the speed was then reduced to 100 rpm. After this, the pre-distribution of the mixture was conducted by spinning the chip at 1000 rpm for 10 s (Fig. 3b-IV). Lastly, by increasing the speed of centrifugation to 4000 rpm valves III were burst open (Supplementary Fig. S2). This allowed the pre-distributed mixture to enter the reaction chamber, where it dissolved the preloaded primers to form a complete LAMP reaction mixture (Fig. 3b-V). After completion of the spin program, thermal control was introduced at 65 °C to initiate LAMP. The reaction time in our experiments was 60 min, and the time needed to complete the entire assay was approximately 70 min.

### Testing for cross-contamination

**Figure 4:**
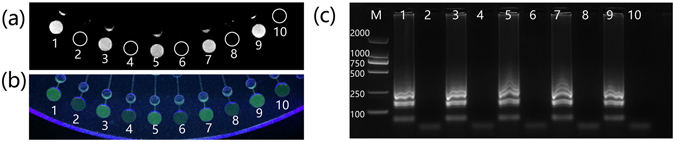
Cross contamination testing of the chip. (**a**) Image captured in the gel imager. Reaction chambers are numbered from 1 to 10, and the dashed lines mark the positions of reaction wells with no signals. (**b**) Photo of signals excited by UVC and taken by a camera. (**c**) Electrophoretogram of the amplicons in each reaction chamber. M represents the DNA marker.

The reaction chambers in the chip were connected by a channel that was filled with air. Evaporation of liquids was inevitable during the heating step at 65 °C^40^, raising the possibility of liquids seeping into adjacent wells. To determine if the evaporation led to cross-contamination of primers, we preloaded primer pairs for *Sty* in odd-numbered reaction chambers and primer pairs for *Sau* in even-numbered reaction chambers. Only *Sty* cells were added to the lysis sample at a final concentration of 100 CFU μL^−1^. All the steps of the analysis for detection of bacteria were performed and gel electrophoresis as well as visual fluorescence detection was used to evaluate the results. In the second method, calcein (25 μM) and manganese (II) chloride (500 μM) were added to the LAMP mastermix before the reaction. Calcein molecules bound with manganese ions remain quenched, and the LAMP solution appears orange initially. As the LAMP reaction proceeds, the pyrophosphate ions generated during the course of the reaction precipitate manganese ions, enhancing calcein’s green fluorescence, which can be observed under ultraviolet and even visible light^42^. After cell lysis, liquid control, and the LAMP reaction, the chip was placed in a gel imager and observed under epi blue excitation light (Fig. 4a). The signals in the different reaction chambers could also be distinguished under UVC by the naked-eye (Fig. 4b). In addition, by peeling off the PSA and pipetting the solutions out, results of the corresponding reaction chambers could also be examined by gel electrophoresis (Fig. 4c). Strong signals were detected in the odd-numbered chambers, and no signals were found in the even-numbered chambers, demonstrating that no cross-contamination of primers occurred between adjacent wells during the reaction.

### LoD for different kinds of bacteria

**Figure 5:**
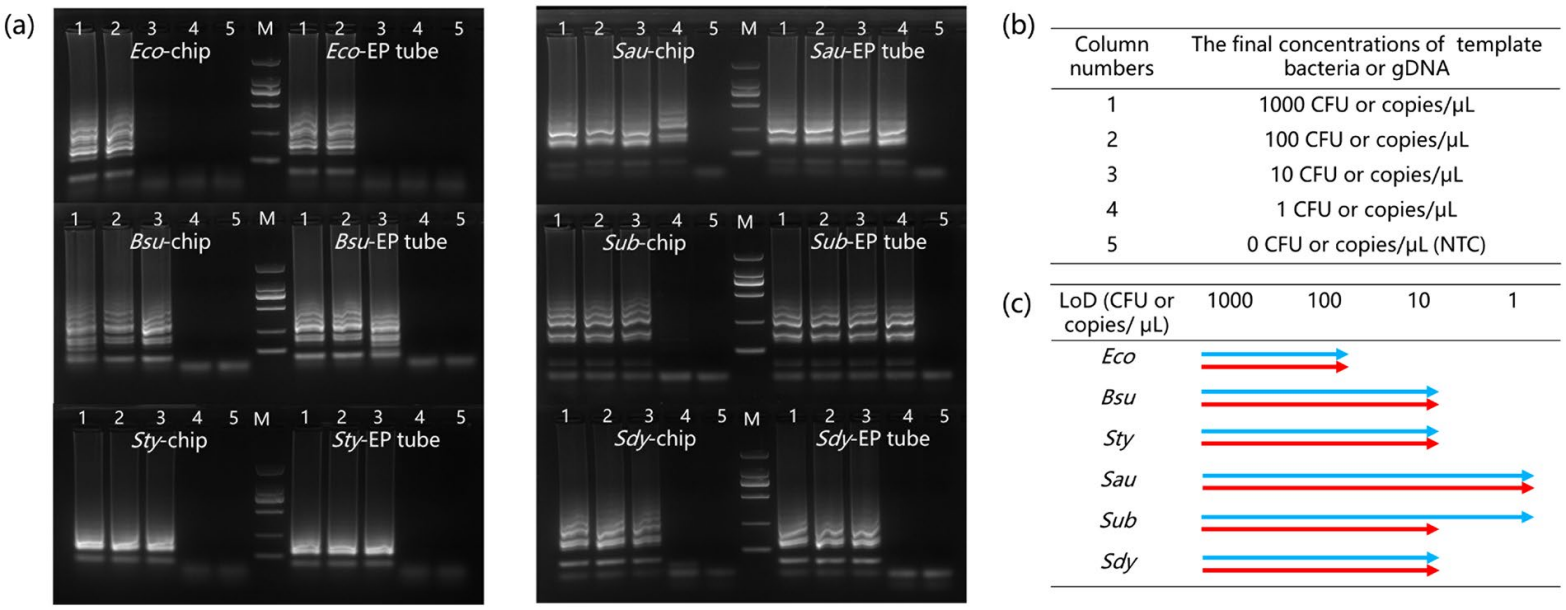
The on-chip and in-tube LoDs of six kinds of bacteria. (**a**) Electrophoretograms of each amplification product with different concentrations of template. Lane marked M represents the DNA marker. The molecular weights of the bands (from the top to bottom) are 2000, 1000, 500, 250, and 100 bps, respectively. (**b**) Concentrations of template in each column. (**c**) Diagram of the on-chip (red arrows) and in-tube (blue arrows) LoD of six kinds of bacteria.

LoD of bacteria is a good measure for evaluation and comparison of the performance of chips developed for identification of bacteria. To characterize the LoD of our on-chip assay, six different kinds of bacteria (*Eco*, *Bsu*, *Sty*, *Sau, Sub*, and *Sdy*) were cultured and diluted using water. Samples containing varying concentrations of bacteria (Fig. 5b) were assayed using the chip. The minimum concentration of bacteria that resulted in a detectable electrophoresis band was regarded as the LoD for that bacteria. Additionally, LAMP reactions were performed in tubes for comparison. The gDNA purified from each kind of bacteria served as the template for these reactions. We assumed that one copy of gDNA equaled one bacterium. The LoD was assessed by gel electrophoresis, and results from on-chip and in-tube assays were compared. Figure 5a and c depict the LoD of the different kinds of bacteria. The on-chip assay detected as low as 100 CFU μL^−1^ (final concentration) of *Eco* and 1 CFU μL^−1^ of *Sau*, respectively, which mirrored the results of the LoD determined by in-tube method. For *Bsu*, *Sty* and *Sdy*, the LoDs were 10 CFU μL^−1^ for the on-chip assay, which were similar to those observed when the amplification was performed in-tube. As little as 10 CFU μL^−1^ of *Sub* were detected with the chip, but the in-tube LoD of *Sub* was lower (1 copy μL^−1^). We attributed this difference to non-specific adsorption of NAs and enzymes to the surface of the PMMA, which was fabricated by machining and whose surface was relatively rough compared to injection molding. Though the off-chip assay demonstrated a lower LoD for *Sub*, the difference was within one order of magnitude. Additionally, no signals were detected for negative template control (NTC) samples. These experiments demonstrated that the chip-based automated assay resulted in a similar performance when compared to the bench-top manual protocol (Fig. 5c). The sensitivity of detection of our on-chip assay could be possibly improved further by modifying the surface of the chip, and/or utilizing the injection molding technique to fabricate chips.

### Specificity of multiplexed detection of bacteria by the chip

**Figure 6:**
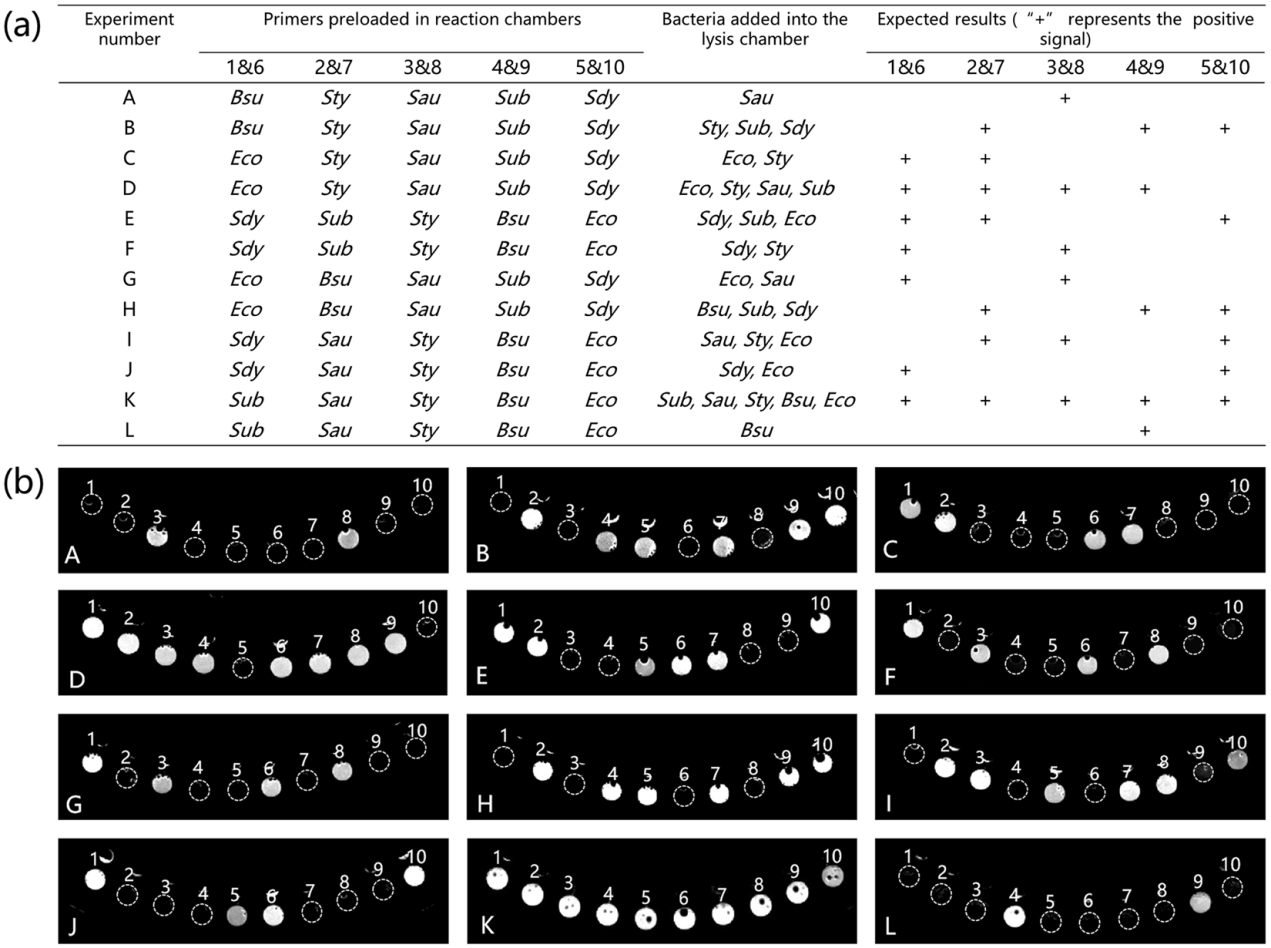
Flexibility and accuracy testing of the chip to detect different combinations of bacteria. (**a**) Design of 12 experiments with different configurations of preloaded primers and different combinations of bacteria. The expected results are also illustrated in the graph. (**b**) Results of each experiment. All reaction chambers were numbered from 1 to 10, and the dashed lines mark the positions of reaction wells with no signals.

Ideally, a chip should be able to detect different types of pathogens rapidly and accurately. We tested our chip for its ability to identify different types of bacteria. Twelve combinations of bacteria spiked in water were prepared as samples to test the performance of our chip. Before introducing the samples in the chip, different primer pairs were preloaded into the reaction chambers. As shown in Fig. 6a, each experiment was designed to lyse different compositions of bacteria with varying configurations of preloaded primers. Theoretically, only the chambers containing corresponding template DNA would show positive signals after the reaction. The final concentrations of all bacteria were uniformly adjusted to 100 CFU μL^−1^ in these experiments. Calcein molecules and manganese chloride were also added to the LAMP mastermix. The results of the analysis were recorded using a gel imager by directly capturing the fluorescence emitted by the chip. These results were compared with the expected results as shown in Fig. 6. Results of experiments A-L were in agreement with the expected signal configurations, indicating that the integrated chip was robust, i.e., the chip was able to accurately identify bacteria. Even though only six kinds of bacteria were tested as models, the current chip design could be upgraded to detect more pathogens in separate reaction chambers on a single chip.

### Real-time detection of bacteria in serum samples

**Figure 7:**
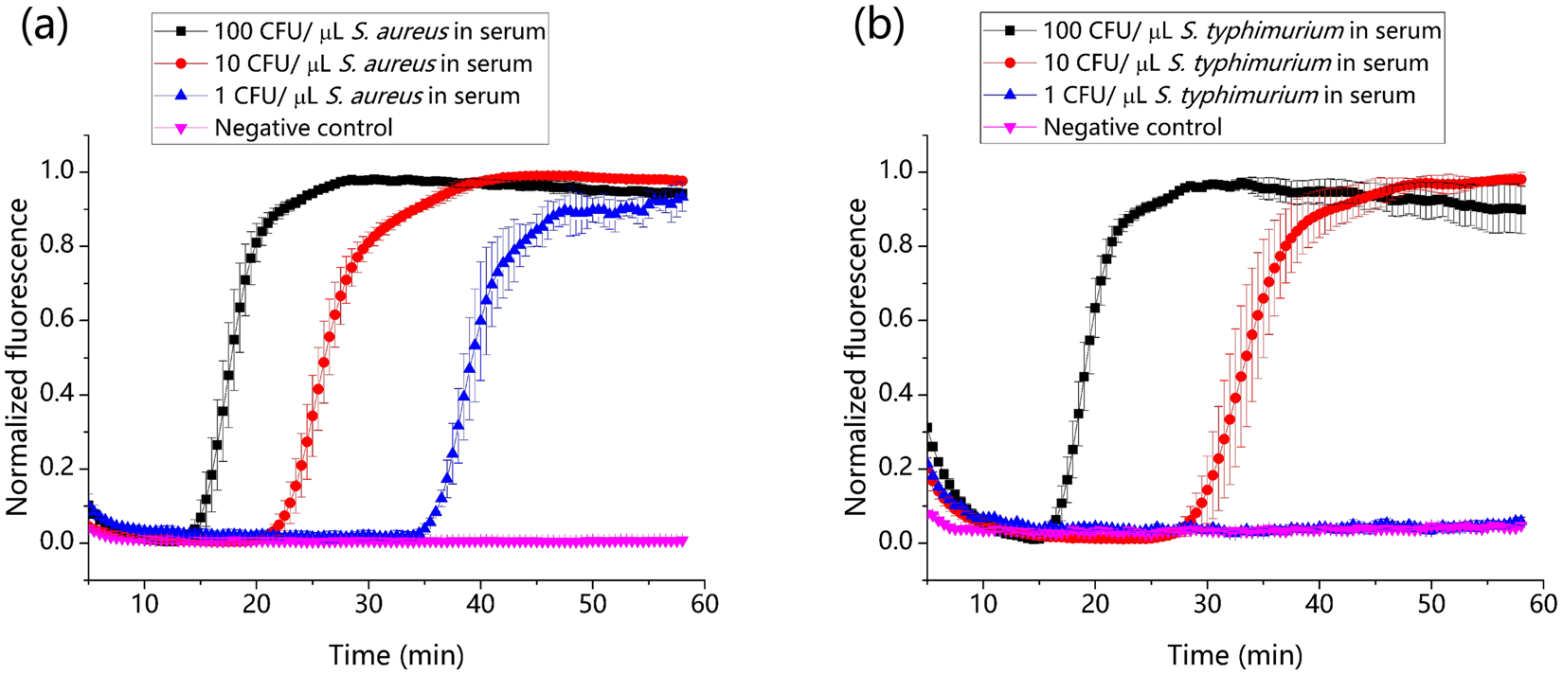
On-chip real-time amplification plot. (**a**) Detection of different concentrations of *S. aureus*. As low as 1 CFU μL^−1^ of *S. aureus* were detected from the serum sample. (**b**) Detection of different concentrations of *S. typhimurium*. Up to 10 CFU μL^−1^ of *S. typhimurium* were detected from the serum sample. The standard deviations of the fluorescence signals of the five reaction chambers used for each pathogen are depicted as error bars in the graphs.

*S. aureus* and *S. typhimurium* are the two major causative agents of infectious diseases such as sepsis. To test the ability of the chip to detect these bacteria in clinical samples, we spiked these bacteria in human serum to mimic clinical samples. Bacteria were purified from these spiked samples and enriched in water as described in materials and methods. The samples were analyzed using on-chip detection process. Reaction chambers numbered 1 to 5 were preloaded with primers for *S. aureus* and chambers numbered 6 to 10 were preloaded with primers for *S*. *typhimurium*. The fluorescence intensities were monitored by real-time fluorescence scanning. As little as 1 CFU/μL of *S. aureus* and 10 CFU/μL of *S. typhimurium* could be detected in these experiments (Fig. 7). These values were similar to the LoDs calculated for these bacteria during analysis using the chip. The sensitivity of our assay could be further increased by using a larger volume of serum and a smaller volume of elution buffer, i.e., by enhancing the enrichment factor and by optimizing the LAMP primer pairs, such approaches could also help avoid false negative results in clinical cases where bacterial loads are lower. Among these approaches, using a larger volume of serum is most direct and effect, and we have performed experiments to demonstrate it. As shown in Supplementary Fig. S7, the LoD of *S. aureus* and *S. typhimurium* were decreased to 0.1 CFU/μL and 1 CFU/μL, respectively. The LoD of *S. typhimurium* is equal to that of gold-standard chemical lysis from blood and PCR done by Syed Riyaz-Ul-Hassan *et al*. (1 CFU/μL)^43^. We could extend the method of capture and enrichment of bacteria from serum samples to bacteria present in urine samples. Thus, the method of sample preparation for on-chip analysis can be used for multiplexed detection of bacteria present in different types of clinical samples.

## Discussion

We have developed a microfluidic chip for the detection of pathogenic bacteria. Our method of detection is rapid, highly sensitive and accurate. Operations like mechanical lysis using beads, clarification of cellular debris as well as LAMP have been integrated seamlessly and are performed in an automated mode on the chip. The complete sealing of the chip by PSA and the high degree of automation have reduced contamination risks and the possibility of human errors. Our chip was robust during experimentation and testing. Different types of bacteria could be detected accurately from water and mimics of clinical samples by coupling up a purification and enrichment protocol with the on-chip analysis.

The chip displayed the following advantages: 1) it was kept stationary during lysis, which simplified the use of valves and, therefore, was easy to fabricate and operate; 2) the time of duration of the entire workflow (~70 min) was shorter than those reported for previously published devices^14, 15^ and, hence this chip has an advantage over others for use in emergency situations; and 3) the amplification results could be detected using an imager, a real-time fluorescence monitor and by the naked-eye, making it versatile in resource-limited areas. Six kinds of bacteria could be detected and identified successfully. More importantly, there was no cross-contamination between adjacent chambers, reducing the risk of false positives. The on-chip assay exhibited LoD values that were equal or similar to those obtained by traditional in-tube LAMP-based method. Further, LoD for *E. coli* observed during analysis by our method was similar to that reported by another LAMP-based centrifugal chip^44^ (2.7 × 10^4^ CFU per 1 mL sample). However, this chip is not equipped to perform bacterial lysis. Czilwik *et al*.^14^ have reported lower LoD value for *E.coli* in their assays (5 CFU in 200 μL serum sample). However, their method uses two rounds of amplification and therefore the time required to complete the analysis was more than 3 times longer than that taken by our chip. Further decrease of LoD for our centrifugal-microfluidics based chip could be achieved by processing a larger volume of serum, modifying the chip surface to avoid non-specific adsorption, and optimizing the LAMP primer pairs.

Current efforts are focused on enhancing the utility of our on-chip analysis method for the detection of bacteria from other kinds of samples such as urine, sputum and river water. Clinical samples usually include blood or sputum, which need to be processed before they can be analyzed on the chip. Efforts are underway to integrate the step of separation of serum or plasma from whole blood^45^, the liquefaction of sputum^46^ and other bacteria capture methods^47, 48^ on the chip itself.

In summary, the prototype of the chip constructed in this study was robust and user-friendly during experimentation. It could accomplish multiplexed detection of bacteria rapidly and accurately. Steps like lysis of bacteria, clarification and LAMP were integrated and could be successfully automated on the chip. Such a chip could serve as a powerful tool in clinical diagnostics for multiplexed detection of bacteria from different samples. Results of the analysis performed using the chip may provide early and crucial information for physicians to conduct life-saving therapies.

## Electronic supplementary material

Multiplex detection of bacteria on an integrated centrifugal disk using bead-beating lysis and loop-mediated amplification

Liquid control on the chip
