# Supplementary material for: Multiplex detection of bacteria on an integrated centrifugal disk using bead-beating lysis and loop-mediated amplification
Source: Sci Rep. 2017 May 3;7:1460. doi: 10.1038/s41598-017-01415-x (PMC5431220; doi:10.1038/s41598-017-01415-x)
Supplement: Supplementary file 1 — Multiplex detection of bacteria on an integrated centrifugal disk using bead-beating lysis and loop-mediated amplification [file 41598_2017_1415_MOESM1_ESM.pdf]

Multiplex detection of bacteria on an integrated centrifugal disk using bead-beating lysis and loop-mediated amplification

He Yan, Yunzeng Zhu, Yan Zhang, Lei Wang, Junge Chen, Ying Lu, Youchun Xu, and Wanli Xing

Supplementary Materials

Figure S1. CAD drawing and geometrical parameters of the chip.

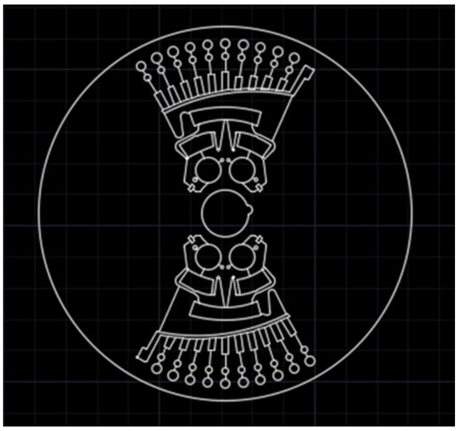

Structure name	Geometrical parameters
The chip has a final dimension of 60 mm (radius) × 3 mm (thickness)	
Inlets/Outlets	radius 0.5 mm, depth 3 mm
Valve I	depth 0.2 mm
Valve II	width 0.1 mm, depth 0.1 mm
Valve III	width 0.3 mm, depth 0.9 mm
Storage chamber I & Lysis chamber	radius 4 mm, depth 1.5 mm
Storage chamber II & Clarification chamber	depth 1.5 mm
Siphon channel I, II, III	width 0.4 mm, depth 0.2 mm
Self-venting channels	width 0.2 mm, depth 0.2 mm
Reaction Chambers	radius 1.5 mm, depth 1.4 mm

Figure S2. Illustration of valves on the chip.

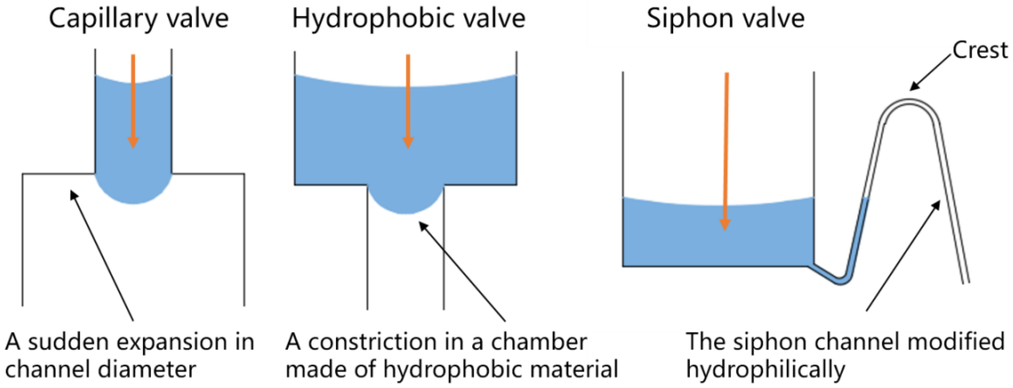

Figure S3. Pre-preparation of spiked serum sample. (a) Diluted serum sample was pushed through a filter and bacteria were captured on it. (b) The filter was washed by water. (c) The syringe was pulled slowly and pure water was drew up and through the filter to suspend bacteria.

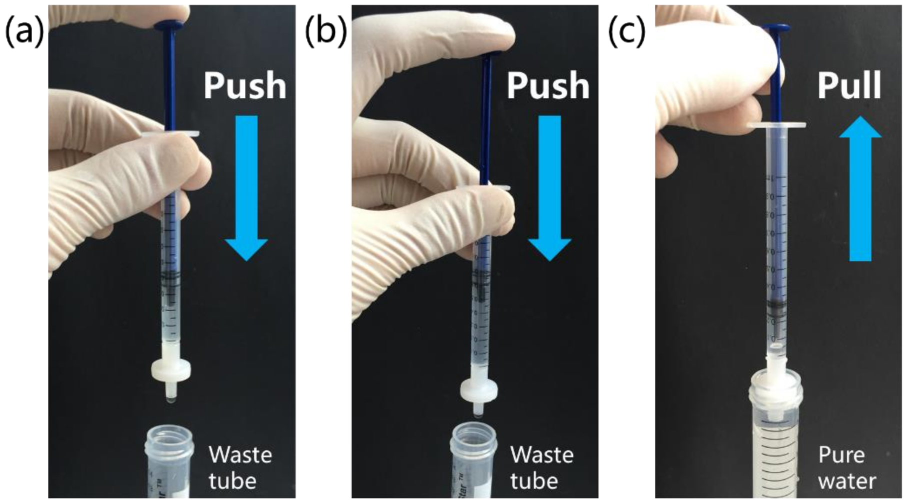

Figure S4. The relationship between voltage and the spin frequency of magnets.

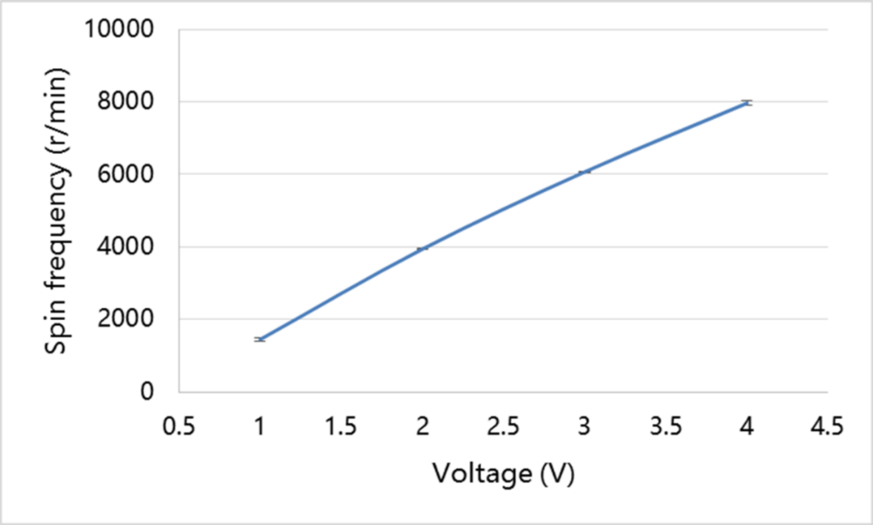

Figure S5. Effect of stirrer voltage on lysis efficiency. Four volts was chosen as the ideal voltage.

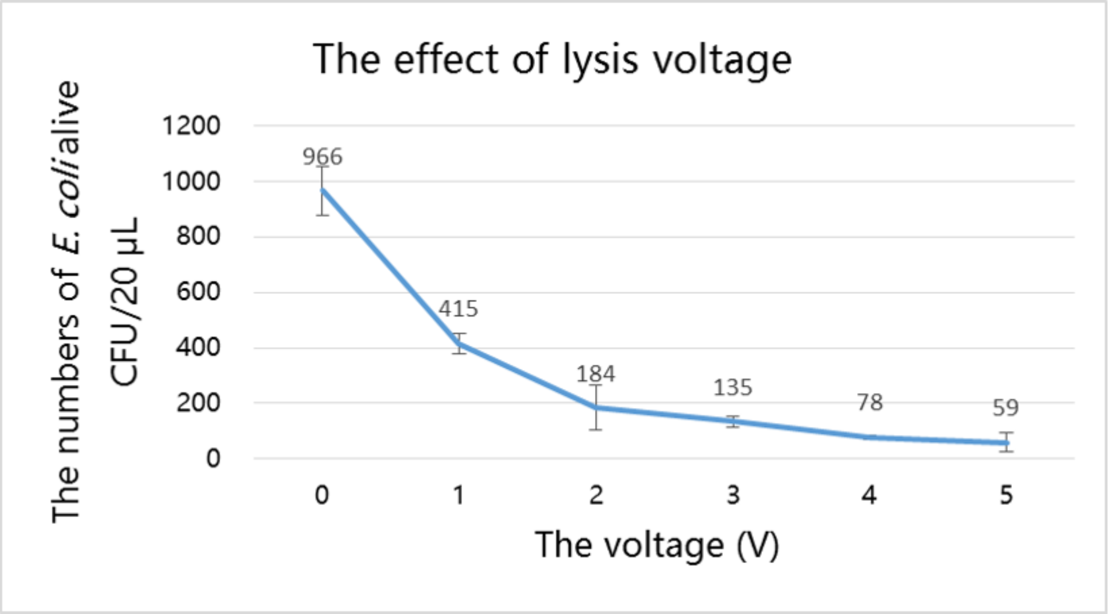

Figure S6. Lysis efficiency of zirconia beads of different mass in the lysis chamber. Two-tenths of a gram of beads was ideal to fill the 100 μL lysis chamber.

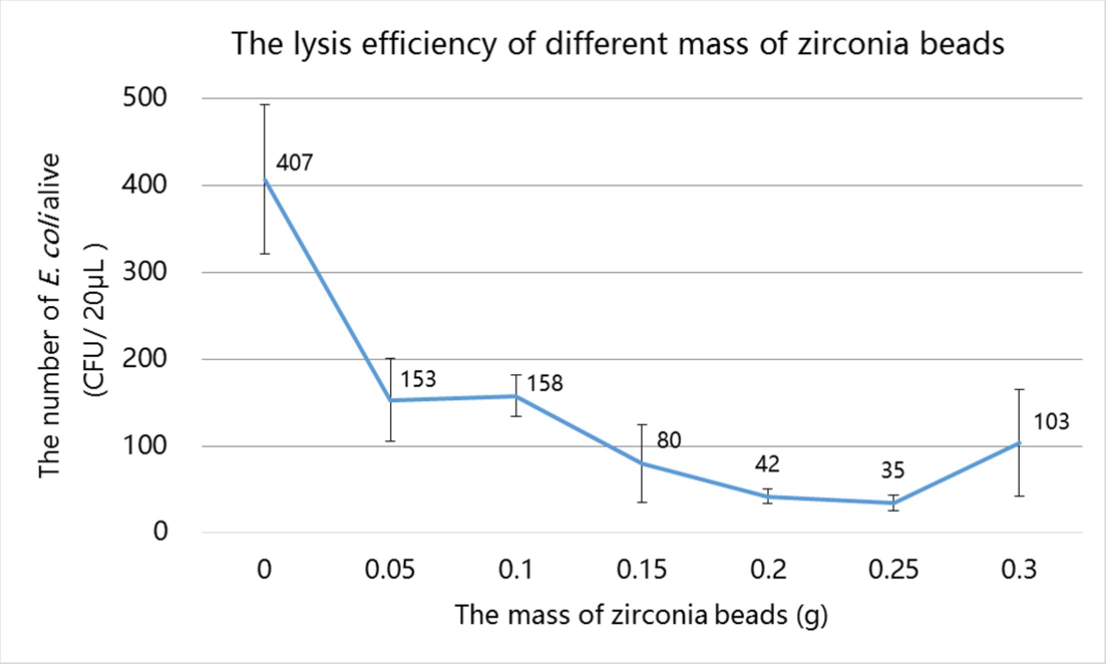

Figure S7. On-chip real-time amplification plot from a larger sample volume. Bacteria were enriched from 1 mL serum and re-suspended in 100 μL water. (a) Detection of 0.1 CFU μL^{-1} of *S. aureus.* (b) Detection of 1 CFU μL^{-1} and 0.1 CFU μL^{-1} of *S. typhimurium*. The standard deviations of the fluorescence signals of the three reaction chambers used for each pathogen are depicted as error bars in the graphs.

(a)

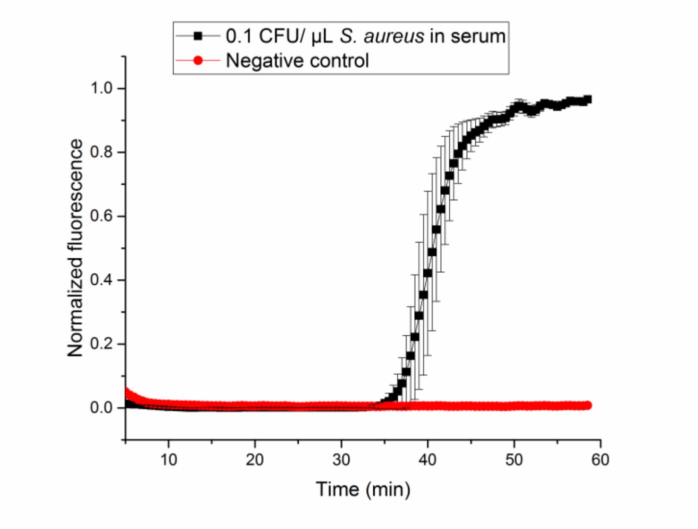

(b)

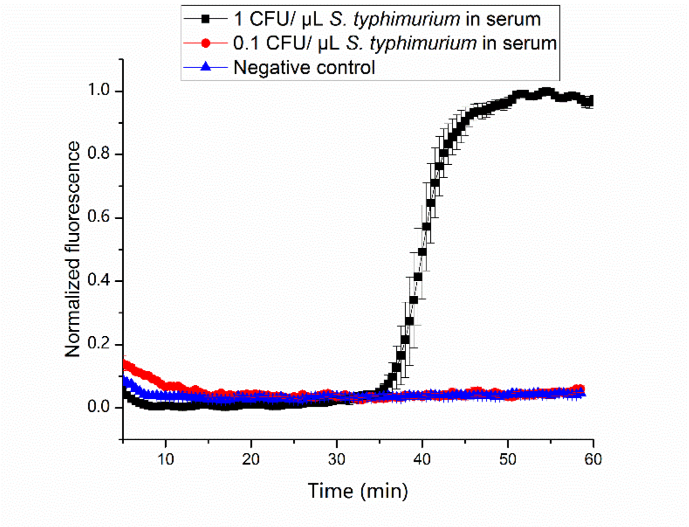

Table S1. Sequences of LAMP primers.

Sau	F3	GCAACTGAAACAACAGAAGC
	B3	TTTTGTGTTGGGCGAGC
	FIP	TCACGGATACCTGTACCAGCATCTCTATGGTCCGAGACCGCAATT
	BIP	GGAACATTTGGATATGAAGCGAGACTGCCATCTTGATTTGTCGTTAC
	LF	TTTCACATACTTAGGTGTTTTGT
	LB	CCAAGTGAAACAAATGCATACAAC
Sub	F3	GCATGTTATTCTCAGAAAACGG
	B3	GCAAAAGCTTTTCTTCTGCA
	FIP	AGTCGCGAATTGAACTTTATACTCATGAAGAACCTATCACTCATCCT
	BIP	TCCACCCACTACCTATTTTTGTAGATTTTTCGAAACTCGTCAGAGG
	LF	ATATTAGCAGTCTCAGT
	LB	CTACGGAGAAAAACATATT
Sdy	F3	GACTGCGCGTGATTCGA
	B3	AGACCAAATCACGGTAAATCC
	FIP	CAATAGCAAATGGCTTCACAATGTTATGGATGTGGTTGCGGGA
	BIP	TATTGGCTCGTATCCGTGCCACTAGGGGTTTTCTTCTCAGAT
	LF	CATCCGCTCCACGGTCTAA
	LB	ATTTTCCGTCGCCAAGATATCG
Sty	F3	GAACGTGTCGCGGAAGTC
	B3	CGGCAATAGCGTCACCTT
	FIP	GCGCGGCATCCGCATCAATATCTGGATGGTATGCCCGG
	BIP	GCGAACGGCGAAGCGTACTGTCGCACCGTCAAAGGAAC
	LF	GGCCTTCAAATCGGCATCAAT
	LB	AAGGGAAAGCCAGCTTTACG
Eco	F3	TGTGGAATTGATCAGCGTT
	B3	TGATTATTGACCCACACTTTG
	FIP	TCTGCATCGGCGAACTGATCGGGTGGGAAAGCGCGTTAC
	BIP	TCTGGTATCAGCGCGAAGTCTAATGAGTGACCGCATCGA
	LF	GCAATTGCCCGGCTTTCTT
	LB	GCAGGCCAGCGTATCGT
Bsu	F3	GCCATATCCGCCAGGTCA
	B3	GCCGAGCAATTCGATGTCAT
	FIP	TCAGGCTGGCTGCTGCGTATACGGAAATGCCAGCAGTGT
	BIP	ATCCATGAAATCGGCGTCCCAACCATTGAGATTGCTTCGCG
	LF	GATGATGGCGCACGATGC

Video. Liquid control on the chip. The video shows the on-chip centrifugation steps after the cell lysis step, sample and mastermix are represented by the red and blue dye, respectively.
